# Moderators of the intention-behaviour and perceived behavioural control-behaviour relationships for leisure-time physical activity

**DOI:** 10.1186/1479-5868-5-7

**Published:** 2008-02-01

**Authors:** Steve Amireault, Gaston Godin, Marie-Claude Vohl, Louis Pérusse

**Affiliations:** 1 Department of Social and Preventive Medicine, Division of Kinesiology, Laval University, Québec (Québec), Canada; 2 Research Group on Behaviour in the Field of Health, Laval University, Québec (Québec), Canada; 3 Department of Food Science and Nutrition, Laval University, Québec (Québec), Canada; 4 Lipid Research Centre, CHUL Research Centre, Québec (Québec), Canada; 5 Canada Research Chair on Behaviour and Health, Faculty of Nursing, Laval University, Pavillon Paul-Comtois, Local 4106, Québec (Québec), G1K 7P4, Canada

## Abstract

**Background:**

Intention is a key determinant of action. However, there is a gap between intention and behavioural performance that remains to be explained. Therefore, the aim of this study was to identify moderators of the intention-behaviour and perceived behavioural control (PBC)- behaviour relationships for leisure-time physical activity.

**Method:**

This was tested in reference to Ajzen's Theory of Planned Behaviour. A sample of 300 volunteers, 192 women and 108 men, aged 18 to 55, participated in the study. At baseline, the participants completed a self-administrated psychosocial questionnaire assessing Ajzen's theory variables (i.e., intention and perceived behavioural control). The behavioural measure was obtained by mail three months later.

**Results:**

Multiple hierarchical regression analyses indicated that age and annual income moderated the intention-behaviour and PBC-behaviour relationships. However, in the final model predicting behaviour (R^2 ^= .46), only the interaction term of PBC by annual income (β = .24, *p *= 0.0003) significantly contributed to the prediction of behaviour along with intention (β = .49, *p *= 0.0009) and past behaviour (β = .44, *p *< 0.0001).

**Conclusion:**

Physical activity promotion programs would benefit not only from focusing on increasing the intention of low intenders, but also from targeting factors that moderate the perceived behavioural control-behaviour relationships.

## Background

Regular physical activity and high physical fitness are associated with numerous health benefits such as reduced risk of premature death from all causes and cardiovascular diseases, type 2 diabetes, specific types of cancer (i.e., colon and breast cancer) and osteoporosis (see Warburton, Shannon and Bredin [1] for review). However, depending on survey methods, it is estimated that only 25% to 40% of the adult population reaches the physical activity level recommended for health benefits [2,3]. This observation highlights the need to devote more attention to factors explaining why some people are active while others remain sedentary.

In the Theory of Planned Behaviour (TPB; Ajzen [4]), considered as one of the most useful theories to study the cognitive determinants of behaviour, intention is a key predictor of behaviour in a wide range of health domains. Reviews and meta-analyses provide empirical support for the predictive power of intention, indicating that it accounts for 20% to 40% of the explained variance of physical activity behaviour [5-7]. According to Cohen [8], this constitutes a medium to large effect size. Nonetheless, there remains a gap between intention and action [9], caused mainly by those who express a positive intention to exercise but do not act; this group represents about one third of the population [10]. This observation is congruent with the analysis of Sheeran [11], who also identified this group as the main source of the lack of consistency between intention and behaviour.

In fact, this gap between intention and behaviour could be attributed to differences in cognitions or other unknown factors. Interestingly, evidence suggests that differences in the intention-behaviour relationship between active and inactive intenders were not attributable to differences in cognition (i.e.: attitude, subjective norm, perceived behavioural control and intention) [11,12]. Thus, other specific factors must be investigated. According to Baron and Kenny [13], the absence or the inconsistence in the presence of a theorized association between an independent variable (e.g.: intention) and a dependent variable (e.g.: behaviour) may indicate that a third variable affects the direction and/or the strength of this relationship. That is, the intention-behaviour might vary according to different levels of a third variable, known as an effect modifier or a moderator. Therefore, one avenue of research to help understand the gap between intention and behaviour is to investigate moderators of this relationship. Indeed, for a given level of moderator, the intention-behaviour relationship should be higher.

The adoption of physical activity depends not only on intention, but also on a variety of control factors such as abilities, resources and conditions. That is, the performance of physical activity is not fully under complete volitional control. According to the TPB [4], the construct of perceived behavioural control (PBC) deals with human behaviour not under complete volitional control. As indicated by Ajzen [14], PBC can be viewed as the combined influence of two components: self-efficacy (ease or difficulty of adopting a behaviour) and controllability (the extent to which the behavioural performance is up to the actor). Within the TPB, perceived behavioural control (PBC) plays different roles in the prediction of behaviour [4]. First, PBC can contribute to the prediction of behaviour along intention (PBC → behaviour). Second, among individuals who express the same level of intention of performing a given behaviour, those with a higher PBC are likely to try harder to perform the target behaviour compared to individuals with lower levels of PBC; this is the intention × PBC interaction hypothesis (intention × PBC → behaviour). However, in order to play these roles, two conditions must be met: 1- the target behaviour must not be completely under volitional control; 2- PBC must reflect accurately "actual" or "real" control over the target behaviour [4]. This latter explanation is congruent with Sheeran, Trafimow and Armitage [15], who for exercise behaviour observed a significant difference between PBC and a proxy measure of actual control among 73% of their study sample (*N *= 226). They reported that when PBC was realistic, PBC explained twice as much variance in behaviour compared to unrealistic PBC. In addition, when PBC was realistic, PBC moderated the intention-behaviour relationship, whereas it did not when PBC was unrealistic. Therefore, investigating PBC as a moderator of the intention-behaviour relationship and scrutinizing moderators of the PBC-behaviour relationship is justifiable.

### Moderators

In scientific literature, PBC has received mixed evidence for its moderating effect of the intention-behaviour relationship [16,17]. About half of the studies reviewed showed a moderating effect of PBC on the intention-behaviour relationship. In each case, higher PBC was associated with better intention-behaviour consistency. In the specific field of exercise/physical activity, four studies have reported a moderating effect of PBC on the intention-behaviour relationship. In the studies by Kimiecik [18] and Payne, Jones and Harris [19], this moderating effect was observed among a sample of 332 and 289 employees, respectively, over a period of two weeks, whereas in the study by Terry and O'Leary [20], this effect was reported among 146 undergraduate students over a period of four weeks. In the fourth study [21], the moderating effect of PBC among two samples of 105 undergraduate students over a period of two weeks was borderline (*p *= 0.075). Another study reported a moderating effect for self-efficacy, a variable close to the concept of PBC [4]. In their study, Courneya and McAuley [22] investigated the moderating role of self-efficacy on the intention-behaviour relationship among 170 undergraduate students. They observed that higher perception of self-efficacy was associated with higher intention-behaviour consistencies at four-week follow-up. Given that Ajzen [14] considers self-efficacy as one of the sub-dimensions (i.e., perceived difficulty) of PBC, the moderating role of self-efficacy on the intention-behaviour relationship was also assessed.

It was also reported that individuals who both anticipated regret and had the intention to practice physical activity showed better intention-behaviour consistency, compared to intenders who did not anticipate regret among four samples (97 ≤ *N *≤ 254) of undergraduate students [23,24]. Anticipated regret is the perceived feeling of regret if the target behaviour is not performed. Among the theoretical reasons why anticipated regret could be a moderator of the intention-behaviour relationship was its association with greater intention stability [23,24]. Intention stability has been clearly demonstrated as one of the most important moderators of the intention-behaviour relationship, including exercise/physical activity [24,25]. Thus, the intention of individuals who anticipate regret for not exercising should better predict behaviour, compared to those not anticipating regret for not exercising.

Moderation effect was reported for past behaviour for both the intention-behaviour [24] and PBC-behaviour [26] relationships. However, two opposite findings were reported concerning the intention-behaviour relationship. On the one hand, some researchers observed that the intention-behaviour relationship was stronger when past behaviour was high. For instance, among a sample of undergraduate students (*N *= 185), Sheeran and Abraham [24] observed that when the frequency of past physical activity was moderate or high, intention was a significant determinant of behaviour whereas it was not when the level of past behaviour was low. The main reason offered for this moderating effect was that past behaviour was related to intention stability (see also Conner and Godin [25]). However, it has also been documented that the intention-behaviour relationship was low when the behaviour had been frequently performed in the past (e.g.: Verplanken, Aarts, van Knippenberg and Moonen, [27]; for travel mode choice). This latter observation is congruent with Triandis' Theory of Interpersonal Behaviour (TIP; Triandis [28]), suggesting that behaviour falls less under the control of cognition when it is performed frequently. Thus, this controversy in findings justifies performing additional tests on the moderating effect of past behaviour on the intention-behaviour relationship.

Concerning the PBC-behaviour relationship, Ajzen [29] stated that past behaviour should moderate the PBC-behaviour relationship. The potential mechanism by which past behaviour may act as a moderator relates to actual control. If individuals are familiar with the behaviour to be adopted, which is likely to be the case for individuals with high levels of past behaviour, PBC should be more accurate and, consequently, PBC should adequately reflect actual control and the PBC-behaviour relationship should be stronger. Notani's meta-analysis [30] provides some empirical support for the PBC × past behaviour interaction in predicting behaviour. Indeed, PBC was a significant determinant of behaviour for samples familiar with the behaviour, whereas it was not a significant determinant of behaviour among samples unfamiliar with the behaviour. In the specific field of exercise/physical activity, Norman, Conner and Bell [26] reported a better PBC-behaviour relationship among patients attending health clinics (*N *= 87) who frequently exercised in the past. When the frequency of past physical activity was moderate or high, PBC was a significant determinant of physical activity whereas it was not a significant determinant when the frequency was low.

Age was found to moderate the intention-behaviour relationship. According to the meta-analyses of Hagger, Chatzisarantis and Biddle [6] and of Downs and Haussenblas [7], a lower intention-behaviour relationship was observed among younger individuals compared to older age groups. In their meta-analyses, Hagger, Chatzisarantis and Biddle [6] found that younger samples from 72 studies (adolescent and college, aged under 25, based on mean age of study samples) showed a significantly weaker intention-behaviour relationship when compared to older samples (aged 25 or older, based on mean age study samples). Similarly, among samples of 111 studies, Downs and Hausenblas [7] reported a weaker intention-behaviour relationship for samples of children/adolescents (aged 8 to 17) compared to samples of young (aged 18 to 25), middle-age (aged 26 to 64) and older (aged 65 or older) adults. They also reported a stronger intention-behaviour relationship for samples of young and older adults compared to middle-age adults. Two explanations have been suggested for the moderating effect of age on the intention-behaviour relationship. First, younger individuals may have unstable intentions and, second, they may have a lack of direct experience with the behaviour compared to older individuals [6,31]. This argument was also suggested in Natoni's meta-analysis [30] to explain why the PBC-behaviour relationship for non-student samples (older individuals) was significant, whereas it was non significant for student samples (younger individuals).

The presence of physical activity facilities (built environment) should also receive more attention due to its possible association with a higher level of physical activity [32]. For instance, perceived access to a low-cost recreation facility in a neighbourhood was reported to moderate the intention-behaviour relationship for walking behaviour [33]. A strong perception of closer access to the facility was associated with a more consistent intention-behaviour association. We consider that the perceived built environment should be viewed as a potential barrier to a behaviour and, therefore, the mechanism by which it can moderate the intention-behaviour relationship could be similar to that of the intention × PBC interaction, as suggested by Ajzen [4]. However, given that this latter study (*N *= 351 adults) was based on a cross-sectional design and used a measure of past behaviour instead of future behaviour, it is advisable to conduct additional tests of its moderating effect.

### Other potential moderators of intention-behaviour and PBC-behaviour relationships

Baranowski, Anderson and Carmack [34] indicated that the effectiveness of physical activity intervention programs based on theoretical models would be increased if study samples were stratified on factors that may moderate model predictiveness. Consequently, new tests for moderation effects of the intention- and PBC-behaviour relationships are needed. In this regard, factors such as annual income and educational level have all been reported to be consistently associated with physical activity (see Trost, Owen, Bauman, Sallis and Brown [35] for review), although there is a lack of information on their potential moderating effect. It is reasonable to surmise that the intention- and PBC-behaviour relationship will be stronger for individuals with higher annual incomes (higher financial resources and access to equipment) and higher levels of education (greater knowledge). In addition, in the field of physical activity, very few studies have attempted to include biological and genetic variables in a conceptual framework. Because physical activity is a biologically-based behaviour, biological and genetic factors could moderate cognition-behaviour relationships [34,36]. As such, the relationship between genetic susceptibility to obesity (familial history) and physical activity should be tested. Indeed, parental overweight/obesity increases an individual's risk of obesity in both childhood and adulthood [37-39]. It is suggested that members of a family share genes and familial environment, including lifestyle and behaviour, leading to obesity, poor nutrition and sedentary activities. In turn, obesity, characterized by high body mass index (BMI), is inversely associated with physical activity level [40,41]. It is likely that genetic susceptibility to obesity assessed by a positive family history of obesity (FHO) may also influence participation in physical activity. Thus, in the context of the present study, the moderating effect of BMI and FHO status was investigated.

In summary, this study aimed at testing psychosocial moderators as well as socio-demographic and biological moderators of the intention-behaviour and PBC-behaviour relationships for leisure-time physical activity.

## Method

### Population and sample

Participants in the present study were part of a larger study on genetic "susceptibility" to obesity. They were recruited in the Quebec City metropolitan area via local newspapers and radio advertisements between May 2004 and March 2007. Also, e-mails were sent to university students and employees as well as hospital and government employees. A trained research assistant conducted a 15-minute telephone interview with people who responded to the advertisements. Candidates were given an appointment at the local university where they completed a series of laboratory tests, including the psychosocial questionnaire on physical activity. Assessment of behaviour at follow-up was obtained by mail three months later. Of the 349 volunteers who completed the psychosocial physical activity questionnaire at baseline, 300 successfully completed the study and were retained for data analysis. All participants signed the consent form of the study approved by the Ethics Committee of the local university.

### Behaviour

Leisure time physical activity was assessed at 3-month follow-up by the following question, previously validated and tested by Gionet and Godin [42] and Godin, Jobin and Bouillon [43]: "Within the last 3 months, how often did you participate in one or more physical activities of moderate intensity, totalling at least 30 minutes in a same day during your leisure time?" The response is reported on the following 7-point scale: Not at all (+1), about once a month (+2), about two or three times a month (+3), about once a week (+4), about twice a week (+5), about three times a week (+6), four or more times a week (+7).

### Psychosocial variables

A definition of the behaviour under study was first presented as a reference for answering the questions. Regular physical activity participation was defined as practising a total of at least 30 minutes of physical activity of moderate intensity (i.e.: with accelerated breathing and heart beat) in the same day at least three times a week during leisure-time. Examples of this type of activities were also provided (e.g., brisk walking, bicycling, swimming, golf (walking), aqua fitness, skating, dancing, skiing, physical fitness, etc.)

Intention was measured at baseline with the following three questions: 1- "I intend to practice regularly one or more physical activities during the next 3 months;" 2- "I will practice regularly one or more physical activities during the next 3 months;" 3- "I will try to practice regularly one or more physical activities during the next 3 months." Scales ranged from very unlikely (+1) to very likely (+5). The mean of the sum of these three items was used as the intention score. Cronbach's alpha coefficient was 0.89. Perceived behavioural control (PBC) was measured at baseline with the following three questions: 1- "For me, to participate in one or more physical activities during the next 3 months would be very difficult (+1) to very easy (+5);" 2- "I think I am able to participate in one or more physical activities during the next 3 months;" 3- "I am confident that I can overcome obstacles that could hamper my participation in one or more physical activities during the next 3 months." Scales for items 2 and 3 ranged from strongly disagree (+1) to strongly agree (+5). The mean of the sum of these three items was used as the PBC score. Cronbach's alpha coefficient was 0.77. The items used to measure each of the other psychosocial variables as well as their psychometric values are presented in Table 1.

**Table 1:** Psychosocial variables and corresponding psychometric values

**Variables**		
During the next 3 months	Measured Scale	Reliability coefficient

**Self-efficacy**	Very unlikely/very likely	.35^a^
I would be able to participate regularly in one or more physical activities even if...		
1-I have little free time		
2-I am not carrying excess body weight		
**Anticipated Regret**	Very unlikely/very likely	.80^b^
During the next 3 months, if I do not participate in one or more physical activities...		
1-This will bother me		
2-I will regret it		
3-I will worry about it		
**Positive Feeling**	Very unlikely/very likely	.52^a^
If I participate in one or more physical activities...		
1-I will be proud		
2-I would be satisfied		
**Moral Norm**	Strongly disagree/strongly agree	.80^b^
1-It is in my principles to participate in one or more physical activities		
2-To participate in one or more physical activities would be acting in accordance with my personal values		
3-It would be against my principles not to participate in one or more physical activities		
**Descriptive Norm**		
1-A lot of people I know participate in one or more physical activities	Strongly disagree/strongly agree	.55^a^
2-Among the 5 persons you know best, how many of them participate in one or more physical activities?	0 person to 5 people	

*Note*. a: Spearman coefficient correlation (two items only). b: Cronbach's alpha coefficient.

### Other variables

Past behaviour was assessed at baseline with the same question used to measure behaviour at 3-month follow-up. The first biological variable, FHO, was assessed as follows: "How many of your first degree biological relatives (father, mother, brother and sister) have been, or are currently, obese?" If at least one relative was reported as obese, participants were asked which one in the family had a history of obesity. The total number of biological brothers and sisters was also reported. Individuals were classified as FHO + if there was one or more first degree biological relatives reported having a history of obesity and as FHO – if there were none reported as obese during the telephone interview. The second biological variable, BMI, was calculated by dividing weight (kg) by height square (m^2^); the latter measure was taken by a trained research assistant during the laboratory visit. Finally, age, gender, educational level, actual civil status and annual income were also assessed at baseline.

### Statistical analysis

Descriptive analysis of the sample was performed (mean, standard deviation and variable distribution). A hierarchical multiple regression was applied to model behaviour. The following sequence was followed: first, the main theoretical variables, intention and PBC, were entered in the model; second, all other psychosocial variables were added; third, external variables that were statistically significant predictors of intention in simple linear regression were added to the model; and fourth, past behaviour was entered. To remain in the model, a given variable had to reach the conventional statistical significance level (*p *< 0.05).

Tests of moderators [13] of the intention-behaviour relationship were performed for perceived behavioural control, self-efficacy and anticipated regret. In addition, all identified external variables were tested as potential moderators of the intention and PBC-behaviour relationships. A three-step hierarchical regression analysis as recommended by Aiken and West [44] was adopted. To provide a clearly interpretable interaction term and to reduce multicollinearity, the variables were standardized [13,44]. At Step 1, behaviour was regressed on intention or PBC. At Step 2, the moderator was added; and finally, at Step 3, the interaction term (intention or PBC × moderator) was added. A moderating effect was detected if the interaction term was statistically significant and if the explained variance (R^2^) was significantly increased (*p *< 0.05). If a moderating effect was detected, simple slopes by levels of moderator were generated and a comparison of the non-standardized betas was assessed. Statistical software used for all analyses was SAS version 9.1 (SAS Institute Inc., Cary, NC).

## Results

The final sample was composed of 192 women and 108 men with a mean age of 37.7 ± 11.2 years. Mean BMI was 27.5 ± 5.7 kg/m^2 ^and ranged from 18.0 to 54.4 kg/m^2^. Participants were highly educated, since 88.0% of them reported having a post-secondary diploma. Annual income was below CAN $20000 for 35.8% of the participants, whereas 33.8% reported an income equal to or over $40 000. About half (53.5%) of the participants lived without a regular partner.

At baseline, half (50.7%) the participants were active at least three times a week during the last three months. The mean intention and PBC scores to be active regularly during leisure-time in the next three months were, respectively, 4.54 ± .69 and 4.09 ± .74 on a five-point scale. With respect to behaviour at 3-month follow-up, the mean score was 5.39 ± 1.57, indicating that, on average, the participants were active about twice a week during the study period. Intention-behaviour and PBC-behaviour correlations were .53 and .44, respectively, (*p *< 0.0001).

For the prediction of behaviour, both intention (β = .98; *p *< 0.0001) and PBC (β = .30; *p *= 0.04) emerged as significant determinants. When past behaviour was added to the previous model, PBC was no longer significant (*p *= .47), whereas intention and past behaviour were the determinants of behaviour (*ps *< 0.0001).

Tests for moderation effects of the intention-behaviour relationship are reported in Table 2. Age (*p *= 0.002), annual income (*p *= 0.03), BMI (*p *= 0.056) and PBC (*p *= 0.01), were significant moderators of the intention-behaviour relationship. Increment in explained variance was observed for all of these moderators; ΔR^2 ^ranged from .01 to .03; *p *≤ 0.05. The intention-behaviour relationship was verified for three levels of age (under 25, 25 to 49 and 50 or older), annual income (less than CAN$ 20000, CAN$ 20000–39999 and CAN$ 40000 or more), and BMI (less than 24.99 kg/m^2^, 25–29.99 kg/m^2 ^and 30 kg/m^2 ^or more). For PBC, the procedure proposed by Aiken and West [44] was used (i.e.: low level: below mean -1 STD; medium level: between mean – 1 STD and mean + 1 STD; and, high level: mean + 1 STD).

**Table 2:** Multiple regression analysis of moderators of the intention-behaviour relationship

Step	Variable entered	Beta			R^2^	Model F	ΔR^2^	ΔF
						
		1	2	3				
1	Intention	.83***	.83***	.85***	.28	116.45***	-	-
2	Age		.09	.10	.28	58.98***	.00	1.36
3	Intention × age			.24**	.31	43.82***	.03	9.95**

1	Intention	.83***	.83***	.85***	.28	116.45***	-	-
2	Annual income		-.03	-.02	.28	57.65***	.00	.16
3	Intention × annual income			.15*	.29	40.47***	.01	4.67*

1	Intention	.83***	.80***	.82***	.28	116.45***	-	-
2	Body mass index		-.23**	-.20**	.30	64.12***	.02	8.75**
3	Intention × body mass index			.16^γ^	.31	44.36***	.01	3.69^γ^

1	Intention	.83***	.67***	.84***	.28	116.45***	-	-
2	PBC		.22*	.25*	.29	60.95***	.01	4.19*
3	Intention × PBC			.14**	.31	43.67***	.02	6.76**

*Note*. PBC: Perceived Behavioural Control. Variables are standardized (Z-scores) and beta coefficients are non-standardized.

*N *= 299 for model including annual income. For all others, *N *= 300.

^γ ^*p *= 0.056. * *p *< 0.05. ** *p *< 0.01. *** *p *< 0.001.

Tests for differences between non-standardized betas (Table 3) indicated that intention was a stronger determinant of behaviour among the older age group compared to the younger group (*p *= 0.003). Individuals with a high annual income had a stronger intention-behaviour relationship compared to those with a lower annual income (*p *= 0.01). However, no significant differences were observed for PBC and BMI between the three levels. Thus, these latter two variables were not retained as moderators of the intention-behaviour relationship.

**Table 3:** Simple slope analysis for the moderators of the intention- and PBC- behaviour relationships

Moderator-Variables	Level of Moderator		
	
	Low	Medium	High
Intention-behaviour relationship			

Age^a^	.82***	1.24***	1.45***
Annual income^a^	.92***	1.38***	1.47***
Body mass index^d^	1.06***	1.24***	1.23***
PBC^d^	1.02***	1.20***	0.73

PBC-behaviour relationship			

Age^b^	.58*	1.09***	.95***
Annual income^c^	.49*	1.07***	1.39***

*Note*. PBC: Perceived Behavioural Control. a = low level is different from high level; b = low level is different from medium level; c = Low level is different from medium and high level; d = no differences between all groups.

* *p *< 0.05. ** *p *< 0.01. *** *p *< 0.001.

Concerning the PBC-behaviour relationship (see Table 4), the only significant moderators were age (*p *= 0.03) and annual income (*p *= 0.001). Increment in explained variance was observed for both moderators; ΔR^2 ^ranged from .01 to .03; *p *≤ 0.05. Simple slope analysis showed that the younger age group had a lower PBC-behaviour relationship compared to the middle age group (*p *= 0.03), but not the older group (*p *= 0.14). For individuals with a lower annual income, the PBC-behaviour relationship was lower compared to individuals with moderate (*p *= 0.02) and high (*p *= 0.0003) annual income.

**Table 4:** Multiple regression analysis of moderators of the PBC-behaviour relationship

Step	Variable entered	Beta			R^2^	Model F	ΔR^2^	ΔF
						
		1	2	3				
1	PBC	.70***	.70***	.70***	.20	73.35***	-	-
2	Age		.02	.02	.20	36.60***	.00	0.07
3	PBC × age			.18*	.21	26.34***	.01	4.87*

1	PBC	.70***	.70***	.71***	.20	73.35***	-	-
2	Annual income		-.05	-.05	.20	36.35***	.00	0.34
3	PBC × annual income			.26**	.23	28.66***	.03	10.85**

*Note*. PBC: Perceived Behavioural Control. Variables are standardized (Z-scores) and beta coefficients are non-standardized.

*N *= 299 for model including annual income. For model including age, *N *= 300.

* *p *< 0.05. ** *p *< 0.01. *** *p *< 0.001.

The final analyses consisted in testing whether the significant moderators and interaction terms contributed independently to the prediction of behaviour. Two sets of analyses were performed: the moderators of the intention-behaviour relationship (Table 5) and the moderators of the PBC-behaviour relationship (Table 6). Concerning the moderators of the intention-behaviour relationship (see Table 5), intention and past behaviour were significant determinants of behaviour in all models (*ps *< 0.0001). Yet, the interaction terms "intention × age" (*p *= 0.004), and "intention × annual income" (*p *= 0.02) were significant predictors of behaviour. For the zero-order model, R^2 ^was .44. For all other models, R^2 ^ranged from .45 to .46.

**Table 5:** Hierarchical multiple regression models of behaviour for different moderators of the intention-behaviour relationship

Variable entered	Models				
	
	1	2a	2b	3a	3b
Intention	.51***	.38***	.38***	.40***	.38***
Past Behaviour	.44***	.43***	.44***	.43***	.43***
PBC	.10	.08	.07	.07	.08
Age		.07		.12	.07
Intention × age		.19**		.14^γ^	.19**
Annual income			-.05	-.10	
Intention × annual income			.15*	.09	

R^2^	.44	.46	.45	.46	.46
Model F	77.57***	49.46***	48.10***	35.54***	49.46***
ΔR^2^	-	.02	.01	.02	.02
ΔF	-	4.53**	3.29*	3.02*	4.53**

*Note*. PBC: Perceived Behavioural Control. Variables are standardized (Z-scores) and beta coefficients are non-standardized.

*N *= 299 for models including annual income due to missing data. For all others, *N *= 300.

^γ ^*p *= 0.08 * *p *< 0.05. ** *p *< 0.01. *** *p *< 0.001.

**Table 6:** Hierarchical multiple regression models of behaviour for different moderators of the PBC-behaviour relationship

Variable entered	Models				
	
	1	2a	2b	3a	3b
Intention	.51***	.54***	.49**	.52***	.49**
Past behaviour	.44***	.43***	.44***	.44***	.44***
PBC	.10	.06	.09	.08	.09
Age		.06		.12	
PBC × age		.16*		.05	
Annual income			-.06	-.11	-.06
PBC × annual income			.24***	.22**	.24***

R^2^	.44	.45	.46	.47	.46
Model F	77.57***	48.61***	43.87***	36.76***	43.87***
ΔR^2^	-	.01	.02	.03	.02
ΔF	-	3.34*	7.05***	4.23**	7.05***

*Note*. PBC: Perceived Behavioural Control. Variables are standardized (Z-scores) and beta coefficients are non-standardized.

*N *= 299 for model including annual income due to missing data. For all others, *N *= 300.

* *p *< 0.05. ** *p *< 0.01. *** *p *< 0.001.

With respect to moderators of the PBC-behaviour relationship, the results (Table 6) indicate that intention and past behaviour were determinants of behaviour in all models (0.0004 ≤ *p *≤ 0.0009 and *p *< 0.0001, respectively). Also, all interaction terms, in their respective models, were statistically significant predictors of behaviour (*p *= 0.02 for "PBC × age" and *p *= 0.0003 for "PBC × annual income"). For the zero-order variable model, R^2 ^was .44, whereas it ranged from .45 to .47 for all other models.

Finally, Table 7 presents the final model for behaviour combining significant predictors and interaction terms of the previous analyses (i.e., Models 3b of the Table 5 and 6). Intention (*p *= 0.0009) and past behaviour (*p *< 0.0001) remained strong determinants of behaviour. In addition, the "PBC × annual income" interaction term (*p *= 0.0003) remained a significant predictor of behaviour; individuals who have a higher annual income are more likely to translate their perceived control into action. The final model explained 46% of the variance in behaviour.

**Table 7:** Final hierarchical multiple regression model for the prediction of behaviour

Variable entered	Models		
	
	1	2	3
Intention	.51***	.37***	.49***
Past behaviour	.44***	.43***	.44***
PBC	.10	.08	.09
Age		.12	
Intention × Age		.11	
Annual income		-.11	-.06
PBC × annual income		.20**	.24***

R^2^	.44	.47	.46
Model F	77.57***	37.23***	50.78***
ΔR^2^	-	.03	.02
ΔF	-	4.69***	7.05***

*Note*. PBC: Perceived Behavioural Control. Variables are standardized (Z-scores) and beta coefficients are non-standardized.

*N *= 299 for models including annual income due to missing data. For all others, *N *= 300.

* *p *< 0.05. ** *p *< 0.01. *** *p *< 0.001.

## Discussion

The main objective of this study was to identify a number of moderators of the intention-behaviour and PBC-behaviour relationships regarding leisure-time physical activity. The moderators of the intention-behaviour and PBC-behaviour relationships were age and annual income. More importantly, however, only one of these moderators remained significant in the final model, namely annual income as a moderator of the PBC-behaviour relationship. In the domain of physical activity, several studies have reported positive associations between physical activity and annual income (see Trost, Owen, Baumen, Sallis and Brown [35] for review). However, to our knowledge, this is the first study to report moderating effects of annual income on the PBC-behaviour relationship.

The PBC-behaviour relationship was higher for individuals with a higher income compared to those with a lower income. Ajzen [4] stated that PBC provides an accurate prediction of behaviour only when individuals have realistic perceptions of control over a given behaviour, meaning when individuals' perception of control matches actual control over behaviour. This explanation is congruent with Sheeran, Trafimow and Armitage [15], who for exercise behaviour observed a significant difference between PBC and a proxy measure of actual control among 73% of their study sample (*N *= 226). They reported that when PBC was realistic, PBC explained twice as much variance in behaviour compared to unrealistic PBC. Thus, in the context of the present study, it can be suggested that individuals who have a higher income are better able to evaluate their true control over behaviour. Consequently, their expression of control is likely better aligned with true control, either because they face fewer barriers or have the ability to overcome such barriers to physical activity.

Although the other moderators did not reach significance in the final model, they were nonetheless significant moderators when considered individually. In this regard, annual income was also a moderator of the intention-behaviour relationship. Again, it can be suggested that individuals with higher income face fewer barriers to leisure-time physical activity and have better resources than individuals with a lower annual income. Thus, individuals with higher annual income present better intention-behaviour consistency than individuals with lower annual income.

Age was another factor found to moderate the intention-behaviour and PBC-behaviour relationships. Two meta-analytic reviews of the TPB in the field of physical activity have tested age as a moderator of the intention-behaviour relationship. Hagger, Chatzisarantis and Biddle [6] found that younger samples (adolescent and college, aged under 25, based on mean age study samples) showed a significantly weaker intention-behaviour relationship compared to older samples (aged 25 or older, based on mean age study samples). Similarly, Downs and Hausenblas [7] reported a smaller intention-behaviour relationship for samples of children/adolescents (aged 8 to 17) compared to samples of young (aged 18 to 25), middle-age (aged 26 to 64) and older (aged 65 or older) adults. Obviously, our results are in agreement with these observations. Among the potential explanations for these results, it may be suggested that older people (i.e., 50 to 55 years in the present study) are more likely to have an established routine (lifestyle) and, consequently, intention is better aligned with their behaviour. Young adults are more likely to deal with unstable living situations (e.g., entering the workplace full time or a new school, meeting new friends, leaving home, living with a new life partner) and may experience more disruption in life, thereby resulting in a lower intention-behaviour relationship. However, given that Downs and Hausenblas [7] have also observed that middle-age adults (aged 26 to 64) had a weaker intention-behaviour relationship compared to older adults (aged 65 or older); additional studies on the moderating role of age are needed.

Concerning the PBC-behaviour relationship, being older (i.e., 50 to 55 years) was associated with greater PBC-behaviour consistency. This observation is similar to the results reported by Notani [30], who showed that the PBC-behaviour relationship was better among non-student samples (older individuals) compared to student samples (younger individuals). To our knowledge, this moderating role of the PBC-behaviour relationship has not been reported previously in the specific field of exercise/physical activity. The potential explanation for this association was discussed earlier with respect to annual income, but taken together, this factor reinforces the assumption that older individuals have a better evaluation of the true control they have on the regular practice of leisure-time physical activity.

From a practical point of view, the results of this study provide useful information to guide the promotion of physical activity. First, intention was found to be a significant determinant of behaviour. This is congruent with Sheeran's [11] meta-analysis showing that on average, the explained variance between intention and behaviour corresponded to a "large" effect size [8]. Thus, education efforts aimed at increasing intention to practice leisure-time physical activities remain necessary to favour the formation of a positive intention and the regular practice of leisure-time physical activity. This, however, should be done in parallel to other strategies concerned with lowering barriers to behaviour adoption and increasing control over adoption of an active lifestyle. Indeed, among specific sub-groups, other actions will be necessary to strengthen the effectiveness of physical activity promotion interventions. For instance, concerning individuals with a lower income, the interventions, in addition to motivational considerations, should be concerned with actual control. Alternatives to usual supervised physical activity programs and facilities should be offered. The promotion of low cost physical activities or "free" access to physical activity programs should be considered by public health authorities. Obviously, each segment of the population would benefit from specific interventions unlikely to suit all individuals. However, the promotion of leisure-time physical activity should combine strategies aimed at both motivational and control dimensions.

To our knowledge, this is the first study to test social structural factors such as educational level and annual income as moderators of the intention-behaviour and PBC-behaviour relationships. To date, most tested moderators were either cognitions or age and gender. However, given the increasing interest towards the contribution of social structural factors (as well as environmental variables), this timely information is necessary. Indeed, much remains to be learned in order to increase our understanding of the conditions and contexts explaining why some individuals are successful in translating their intentions into action, while others are not successful in this task.

Some limitations in the present study should be acknowledged. First, our study was conducted among a group of well-educated volunteers. Such individuals are likely to have higher intentions and levels of physical activity compared to the general population. Secondly, the non-optimal distribution of intention/PBC and some moderator variables (dichotomous variables like FHO and educational level) may have compromised the power of detection of some moderators, making detection of other moderators more powerful [45]. Thirdly, one of the biological variables, FHO, was based on the subjective evaluation of the participants. This may have compromised its true role as a moderator. Nonetheless, given that two studies reported a good reliability of an individual's report of family weight and height [46,47] we have confidence that our results were not affected by this method of measurement. Fourth, only leisure-time physical activity was studied. It is possible that other aspects of physical activity (e.g., transport) would have identified other moderators. Finally, the measure of physical activity was self-reported; however, we used a validated physical activity questionnaire [42,43].

## Conclusion

Intention and past behaviour were strong determinants of behaviour. Some moderators of the intention-behaviour and PBC-behaviour relationships were identified. In particular, the interaction between PBC and annual income remained a significant determinant of leisure-time physical activity, along with intention in the final model. Thus, physical activity promotional programs would benefit from both increasing the motivation of low intenders and reducing the negative impact of factors that moderate the intention-behaviour and perceived behavioural control-behaviour relationships.

## Competing interests

The author(s) declare that they have no competing interests.

## Authors' contributions

SA coordinated and performed the acquisition of data, performed the statistical analysis and the interpretation of data and drafted the manuscript. GG helped to the data analysis and to the data interpretation. GG, MCV and LP conceived the study design and provided critical review of the manuscript. All authors approved the final version of the manuscript.
